# Complementary and Alternative Medicine Education for Medical Profession: Systematic Review

**DOI:** 10.1155/2012/656812

**Published:** 2012-04-30

**Authors:** Nana K. Quartey, Polly H. X. Ma, Vincent C. H. Chung, Sian M. Griffiths

**Affiliations:** School of Public Health and Primary Care, Chinese University of Hong Kong, Hong Kong

## Abstract

*Purpose*. To help integrate traditional, complementary and alternative medicine (TCAM) into health systems, efforts are being made to educate biomedical doctors (BMD) and medical students on TCAM. We systematically evaluated the effect of TCAM education on BMD and medical students' attitude, knowledge, and behavior towards TCAM utilization and integration with biomedical medicine. *Methods*. Evaluative studies were identified from four databases. Methodological quality was assessed using the Medical Education Research Study Quality Instrument (MERSQI). Study outcomes were classified using Kirkpatrick's hierarchy. *Results*. 3122 studies were identified and 12 studies of mediocre quality met inclusion criteria. Qualitative synthesis showed usage of diverse approaches including didactic, experiential learning, varying length, teacher background and intensity of exposure. More positive attitudes and improved knowledge after intervention were noted especially when teachers were BM trained. However, few studies assessed behavior change objectively. Finally, longer-term objective outcomes such as impact on patient care were not assessed. *Conclusions*. Lack of use of objective and reliable instruments preclude firm conclusion on the effect of TCAM education on study participants. However, positive changes, although mostly subjectively reported, were noted in most studies. Future evaluation should use validated or objective outcome assessments, and the value of using dual trained instructors.

## 1. Introduction

It is estimated that over 80% of the populations of low-income countries are using herbal medicine as part of basic healthcare [1], and the use of traditional, complementary, and alternative medicine (TCAM) is also increasing in high-income countries [2]. In response to the relevance of TCAM in primary health care worldwide, the World Health Organization (WHO) has repeatedly reconfirmed its support in TCAM development in the past decade [3]. In 2008, the WHO Congress of Traditional Medicine promulgated the *Beijing Declaration*. It regards the formulation of TCAM policies as a responsibility of governments worldwide and has reemphasized the importance of integrating TCAM amongst the member states' health system. One of the major policy directions is to strengthen the communication between biomedical doctors (BMDs) and TCAM providers (TCAMP), as well as to develop appropriate TCAM training programs for BMD and medical students [4].

Echoing advocacy of the WHO, the US Institute of Medicine has recommended the introduction of TCAM education for medical professionals at both undergraduate and postgraduate levels [5]. The goals of TCAM education were to equip BMD and medical students with the following competencies: (i) know how to ask patients about their use of TCAM; (ii) be familiar with the most commonly used forms of TCAM so BMD can discuss these practices with their patients; (iii) be able to refer interested patients to reliable sources of information; (iv) know how to obtain reliable information about the safety and efficacy of TCAM [6]. Evaluators of TCAM education have highlighted the difficulties in examining the effects of initiatives in changing BMD and medical students' attitude, knowledge, behavior, and skills related to TCAM [7], and so far a systematic review on the topic has not been published [8].

## 2. Objective

The objective of this review is to examine studies that provide evidence of effective TCAM education. We will describe how these programs influenced the attitude, knowledge, and behavior amongst BM students or clinicians regarding TCAM use amongst patients, as well as the integration of TCAM and BM.

## 3. Methods

### 3.1. Criteria for Considering Studies for This Review

#### 3.1.1. Types of Studies

Randomized controlled trials (RCTs), nonrandomized controlled trials (non-RCTs), and before and after studies.

#### 3.1.2. Types of Participants

Biomedically trained doctors or medical students.

#### 3.1.3. Types of Interventions

Any educational interventions focused on TCAM. These included didactic, experiential, and web-based learning, or any combination of these pedagogical approaches. There was no restriction on the mode of delivery.

#### 3.1.4. Types of Outcome Measure

We included five levels (2a–4b) of educational outcomes described in the modified Kirkpatrick hierarchy. Level-one outcome was excluded in this review as it is only related to the level of satisfaction of TCAM learning experience. Descriptions of these 5 levels of outcomes are as follows [9–11].

- (Level 2a) Modification of attitudes or perceptions: changes in the reciprocal attitudes or perceptions between participant groups towards patients' use of TCAM, as well as the nature of TCAM interventions.
- (Level 2b) Modification of knowledge and skills: for knowledge, this relates to the acquisition of concepts, procedures, and principles related to TCAM; for skills, this relates to the acquisition of problem solving and social skills in handling patients' TCAM use decision or behavior.
- (Level 3) Behavioral change: documents the transfer of learning new knowledge and skills related to TCAM to the workplace or the learners' willingness to apply them.
- (Level 4a) Change in organizational practice: wider changes in the organization or delivery of care related to TCAM, attributable to an educational program.
- (Level 4b) Benefits to patient or clients: any improvement in the health and wellbeing of patients and clients as a direct result of TCAM education programs.

### 3.2. Search Method for Identification of Studies

We searched four electronic databases for primary studies since their inception till Nov 2010: MEDLINE, CENTRAL, EMBASE, and AMED. Search strategies are listed in the appendix. Reference lists of included studies were screened for potentially relevant studies.

### 3.3. Data Collection and Analysis

#### 3.3.1. Selection of Studies

Two investigators (N. K. Quartey and P. H. X. Ma) independently screened all citations generated from electronic search by examining their titles and abstracts. Full texts of potentially relevant studies were then retrieved, and their eligibility was confirmed according to inclusion criteria described above. Discrepancies between the two reviewers were resolved through discussion. Remaining disagreements were handled by a third reviewer (V. C. H. Chung).

#### 3.3.2. Data Extraction and Management

Two reviewers (N. K. Quartey and P. H. X. Ma) independently extracted the following information from included studies using a standardized data extraction form: study design, setting, characteristics of the participants and educational interventions, study duration, and results for all outcome measures.

#### 3.3.3. Assessment of Methodological Quality

Methodological quality of included studies was assessed independently by two reviewers (N. K. Quartey and P. H. X. Ma) using the Medical Education Research Study Quality Instrument (MERSQI) [12]. The MERSQI has 6 main domains; study design, sampling, types of data, validity of evaluation instrument, data analysis, and outcomes. These domains were further divided to yield 10 items, each with an item score ranging from 0 to 3. Hence, each of the 6 domains has a maximum score of 3, giving a total possible score of 18 for each study. Discrepancies in scoring between the two reviewers were resolved by discussion with a third reviewer (V. C. H. Chung).

#### 3.3.4. Data Analysis

As wide variations in characteristics of participants, intervention, outcomes, study quality, and settings were expected, we did not plan to pool results using meta-analysis [13]. Instead, we conducted a qualitative synthesis of findings from included studies [14].

## 4. Results

### 4.1. Search Results and Characteristics of Included Studies

A total of 3122 studies were identified in our electronic search. After scrutiny of the titles and abstracts, we identified 10 potentially relevant articles. We obtained 2 further studies from reference lists of these articles and finally included 12 studies. Characteristics of included studies are listed in Table 1. Amongst the 12 included studies, two were RCTs, one was nonrandomized controlled trial (non-RCT), and nine were single group before and after studies. Nine of the 12 studies were conducted in the United States, two in Israel, and one in the UK (Table 1).

### 4.2. Characteristics of the Participants

Two studies included both residents and senior doctors, and, respectively, two and one targeted at senior doctors and residents only. Two invited both residents and medical students to participate, and the remaining five included medical students only (Table 1).

### 4.3. Characteristics of the Intervention

Two studies examined the effect of a single session of TCAM education [15, 16]. Four studies evaluated weekly educational session ranging from 10 to 15 weeks [19, 20, 22, 25]. One study investigated the effect of 3-year sequential interventions in a cohort of medical students [23]. Five studies did not describe the duration and frequency of interventions [17, 18, 21, 24, 26]. In three studies, teaching was delivered by both biomedical doctors and TCAM practitioners [19, 20, 22]. Respectively, one study involved only biomedical doctors [21], TCAM practitioners [26], and dual trained clinicians [25] in teaching. In one study, both TCAM practitioner and dual trained clinicians delivered the course [15], and one study involved biomedical doctors as well as standardized patients [16]. Two studies utilized web-based teaching [17, 18] and the remaining two did not report teachers' professional background [23, 24].

Three studies delivered didactic lectures [21, 22, 24], and two studies evaluated the effect of experiential learning exclusively [20, 26]. Four examined the effect of a mixed didactic and experiential approach [15, 19, 23, 25]. Range of experiential exposures included (i) enabled participants to take part in yoga, massage, Tái Chi, Rekki, meditation, and acupuncture, (ii) provided live demonstrations of TCAM performed on patients, as well as (iii) demonstrated patients' consultation on TCAM utilization. Standardized patients were used in only one study to simulate TCAM-related clinical encounter [16]. 

### 4.4. Characteristics of Outcome Measures

Classified according to Kirkpatrick's Model, six studies described modification in attitude or perceptions on TCAM (Level 2a), while six reported changes in knowledge or skills (Level 2b). Five studies documented change in clinical behavior related to TCAM (Level 3), but these outcomes were self reported by the participants only. None of the included studies reported changes in organizational practice (Level 4a) or patient outcome (Level 4b).

### 4.5. Methodological Quality of Included Studies

Total MERSQI scores amongst the 12 studies (Table 2) ranged from 8.5 to 13.5, with a mean of 10.83 (standard deviation (SD): 1.79). Mean domain scores were highest for type of data (1.83, SD: 1.03) and study design (1.79, SD: 0.58). Mean scoring were lowest for validity of evaluation instruments (0.36, SD: 0.49) and sampling (0.90, SD: 0.47).

### 4.6. Effects of TCAM Education

#### 4.6.1. Impact on Self-Reported Behavior or Behavioral Intents

**(1) Practicing Physicians**

One RCT reported that an 8-hour TCAM education program had increased cardiologists' likelihood in recommending energy therapies (*P* = 0.001), manipulation (*P* < 0.001), mind body interventions (*P* < 0.001), and therapies originated from other medical systems (*P* = 0.003) compared to cardiologists in the control group. Nevertheless, the exact count was not reported [15]. In a before and after evaluation of a 14-session TCAM for cancer course for family physicians, only 2 participants answered “sometimes” or “usually” when they were queried on their frequency of discussing TCAM with cancer patients at baseline. In similar vein, only 3 reported “sometimes” or “usually” when they were queried on TCAM referral frequency. These figures increased to 16 and 11, respectively, after the intervention. However, statistical tests were not performed to evaluate such difference [19]. In another before and after evaluation of a problem-based TCAM education initiative amongst family practice residents and specialists (breakdown not reported, *n* = 12), eight participants reported higher TCAM referral frequency, and 6 stated more frequent communication with TCAM practitioners after the program [20].

**(2) Residents and Medical Students**

One nonrandomized controlled trial mentioned that residents and senior medical students (breakdown not reported) enrolled in a web-based course on TCAM were more likely to discuss TCAM with patients, as well as to refer patients to TCAM compared to baseline (number of participants discussed TCAM: baseline: 42, 3 months after enrollment: 52, *P* = 0.015). However, followup results for control group were not reported [17].

Finally, in an evaluation of a 10-week elective TCAM course offered to junior medical students, Halterman-Cox et al. reported negative effect of teaching on intentions to refer or provide herbal medicine, massage, prayer therapies, and therapeutic touch to patients [22].

#### 4.6.2. Impact on Knowledge and Skills

**(1) Practicing Physicians**

Results from the RCT conducted by Hewson et al. indicated that cardiologists in the intervention group scored significantly higher in knowledge on the range of TCAM therapies offered by alternative medical systems (*P* = 0.007), energy therapies (*P* < 0.001), manipulative therapies (*P* < 0.001), and mind body intervention (*P* = 0.005). They also demonstrated higher knowledge level on existing support for integrating TCAM medical systems (*P* = 0.007) and manipulative therapies (*P* < 0.001) with biomedicine. In addition, higher knowledge on the existence of trustworthy research in manipulative therapies (*P* = 0.007) and mind body interventions (*P* = 0.004) was also mentioned [15]. The before and after study conducted by Ben-Arye et al. highlighted that participants had became more skillful in referring patients to TCAM [20]. Another study evaluating the effect of web-based educational intervention with similar design had reported significant improvement in junior physicians' performance in a written test on knowledge related to dietary supplements [18]. Finally, a pre- and postevaluation, case-based TCAM tutorial for residents in various specialty was found to be effective in improving residents' knowledge on herbal medicines. The effect was found to be stronger amongst family medicine physicians [24].

**(2) Medical Students**

One RCT reported that TCAM workshop using standardized patients had improved senior medical students' performance in answering multiple choice (*P* < 0.001) and open-ended questions (*P* < 0.001) related to TCAM. Furthermore, compared to students in the control group, workshop participants demonstrated superior skills in handling TCAM-related clinical scenarios (*P* < 0.001), taking TCAM use history (*P* < 0.001), and providing counseling on TCAM use (*P* < 0.001) [16]. In the nonrandomized controlled study conducted by Cook et al., senior medical students in the intervention group scored significantly higher in a TCAM knowledge written test compared to those in the control group (*P* < 0.001). This result remained significant after adjusting for baseline attitudinal difference [17]. Finally, the pre- and postevaluation conducted by Lu and Torres-Harding showed positive effect of a sequential TCAM course in preparing students for counseling patients on the use of acupuncture [23].

#### 4.6.3. Impact on Attitude and Perceptions

**(1) Practicing Physicians**

Results from the RCT conducted by Hewson et al. indicated that cardiologists in the intervention group scored significantly higher in the “openness to CAM” (*P* < 0.001) and “importance of doctor patient relationship and self awareness” (*P* = 0.004) domains of the validated Integrative Medicine Attitude Questionnaire [15].

**(2) Medical Students**

Significant improvement in self-efficacy towards TCAM clinical issues was observed amongst medical students in the nonrandomized controlled study conducted by Cook et al. These improvements relate to discussing TCAM therapies with patients (*P* < 0.001), querying patients' use of TCAM (*P* < 0.001), recognizing the role of TCAM in providing holistic care (*P* < 0.001), and finding clinical evidence related to TCAM. However, these improvement were evaluated by a before- and after-comparison amongst participants only, and comparison with control groups was not reported [17]. In another before- and after-evaluation of an interactive module of TCAM for medical students, improvement in self-efficacy in apprising TCAM clinical trials (*P* < 0.0001) as well as appropriateness of integrating TCAM (*P* = 0.015) were also reported [21]. In another pre- and postinvestigation on the effect of TCAM placement on medical students, Donald et al. reported a significant reduction of skepticism towards TCAM (*P* < 0.001) [26]. With similar design, evaluation of a 3-week elective TCAM rotation conducted by Torkelson et al. highlighted significant improvement in medical students' attitude and self perceived knowledge on TCAM [25]. Finally, the pre- and poststudy conducted by Halterman-Cox et al. reported a negative association between TCAM teaching and general attitude towards TCAM amongst junior medical students. Stronger postcourse nonacceptance was observed amongst modalities taught by TCAM practitioners. These included chiropractic, massage, prayer therapies, therapeutic touch, and nutritional supplements. On the other hand, modalities taught by medical doctors, including acupuncture and herbal medicine, received higher acceptance rating postcourse [22].

## 5. Discussion

### 5.1. What Is Already Known on This Topic?

The addition of TCAM elements in biomedicine curricula is gaining popularity worldwide. In the United States, the National Institutes of Health National Center for Complementary and Alternative Medicine had awarded 14 health profession schools with five-year grants for developing TCAM educational programs [6]. In the UK, the General Medical Council requires medical school to educate students on TCAM and 18 out of 31 surveyed medical school has incorporated TCAM in their curricula [27]. In Canada, a national initiative to integrate TCAM elements in undergraduate medical education has been launched [28]. Similar trend has been observed in Australia [29] and Germany [30]. However, the effectiveness of these programs has not been summarized.

### 5.2. What This Study Adds?

This systematic review identified 12 studies examining the impacts of TCAM educational program on BMD and medical students at various educational outcome levels. Several general insights on the design of future TCAM curriculum can be drawn from these results. Firstly, although all TCAM education programs were designed to enhance BMD and medical students' competency in handling clinical scenarios related to TCAM, the content, duration, and pedagogical approach of interventions amongst the studies were heterogeneous and lacked a common core. Thus, future studies should focus on developing a harmonized curriculum by comparing the effectiveness of different approaches. Secondly, amongst all included studies, the only one that reported negative results has commented on the potential disadvantages of using TCAM practitioners as instructors. This observation echoes findings from studies on the interprofessional collaboration between BMD and TCAM practitioners, in which BMD trained in TCAM are often better accepted than clinicians trained only in TCAM [31]. However, the potential benefit contributed by a dual trained clinician requires further affirmation. A previous systematic review has already pointed out that the design of interprofessional education is often difficult in general and the critical elements for success are yet to be discovered [32].

### 5.3. Suggestions for Future Research

While the methodological quality of included studies did not fare worse than other educational studies in the field [33–35], mediocre methodological quality of included studies has precluded the possibility of drawing a firm conclusion. Methodological limitations are particularly severe in the choice evaluation instruments, as reflected in the low-mean MERSQI domain score of 0.36  out 3. Changes in attitude and skills are popular outcomes amongst included studies and their measurements require the use of reliable instruments. Nevertheless, we only located one study [15] that had assessed attitudinal change with the validated Integrative Medicine Attitude Questionnaire [36–38].

On behavioral outcomes, our overall results indicated that TCAM education lead to more frequent referral to, as well as active discussion on, TCAM with patients amongst BMD and medical students. However, none of the included studies has documented behavioral changes using gold standards (e.g., checking referral and discussion detail from patients' record). Hence, the causal link between TCAM education and improvement in competency is yet to be confirmed. In addition, only one study evaluated TCAM skill acquisition by direct observation of students' performance in managing standardized patients [15]. Future assessment would benefit from using validated tools like the Mini Clinical Evaluation Exercise [39], but relevant adaptations are required.

Finally, we located no studies discussing how TCAM education may change the organization of clinical practice or impact patient outcomes. Future studies will need to focus on these and other outcome measures to elucidate the impact of TCAM education on improving service responsiveness to patients' choice for TCAM. Beyond internal validity, the generalizability of findings included in this systematic review also warrant attention. As the majority of studies were conducted in the U.S., the transferability of their experience to other health systems should be carefully scrutinized.

## 6. Conclusion

Education on TCAM for western doctors appears to be effective in improving attitudes, knowledge, and skills amongst both physicians and medical students towards the use of TCAM. Nevertheless, a solid conclusion cannot be reached due to the methodological limitations of existing studies. Future research using validated outcome instruments as well as patient outcome as endpoints will provide a firmer answer on the impact of TCAM education. In addition, innovations in educational approaches, particularly the role of dual trained clinicians in delivering quality TCAM education, should be further explored within medical educational settings.

## Figures and Tables

**Table 1:** Characteristics of included studies.

First author and publication year	Country and type of participants	Study design	Number of participants in the intervention group	Number of participants in the control group	Type of educational approach	Duration and frequency of intervention	Instructors' professional background	Outcomes reported according to Kirkpatrick level		
								2a: Attitude or perception	2b: Knowledge or skill	3: Behavioral change or intention to change
Hewson et al. 2006 [15]	U.S. BMD	RCT	Enrolled at baseline: 24 completed followup: 20	Enrolled at baseline: 24 completed followup: 16	Didactic plus experiential: didactic lectures (3 hours) and experiential workshops (5 hours)	8 hours, 1 session only	TCAMP, Clinicians trained in both BM and TCAM	X	X	X

Hoellein et al. 2009 [16]	U.S. Medical students	RCT	Enrolled at baseline: 92 completed followup: 92	Enrolled at baseline: 94 completed followup: 91	Didactic plus experiential: TCAM workshop and role play with standardized patients	4 hours, 1 session only	BMD		X	

Cook et al. 2007 [17]	U.S. BMD and medical students	Non-RCT	Enrolled at baseline: 89 completed followup: 79	Enrolled at baseline: 34 completed followup: 34	Web-based: cased base tutorial on TCAM delivered online	Not reported	N/A	X	X	X

Ashar et al. 2008 [18]	U.S. BMD	Single group before and after study	Enrolled at baseline: 335 completed followup: 335	N/A	Web-based: case-based interactive curriculum delivered online	Not reported	N/A		X	

Ben-Aryee and Frenkel 2004 [19]	Israel BMD	Single group before and after study	14 of the 18 participants answered the precourse questionnaire, and 16 answer the postcourse questionnaire	N/A	Didactic plus experiential: experience sharing by TCAMP, case study and experiential workshop	14 weekly sessions, duration of each session not reported	BMD, TCAMP			X
Ben-Aryee et al. 2006 [20]	Israel BMD	Single group before and after study	Enrolled at baseline: 12 completed followup: 12	N/A	Experiential: clinical case presentations	12 weekly session, duration of each session not reported	BMD, TCAMP		X	X

Donald et al. 2010 [26]	U.K. Medical students	Single group before and after study	Enrolled at baseline: 40 completed followup: 29	N/A	Experiential: personal exposure to TCAM modalities during placement	Not reported	TCAMP	X		

Forjuoh et al. 2003 [21]	U.S. Medical students	Single group before and after study	Enrolled at baseline: 67 completed followup: 54	N/A	Didactic: Lectures during core clerkship	Not reported	BMD	X		

Halterman-Cox et al. 2009 [22]	U.S. Medical students	Single group before and after study	Enrolled at baseline: 52 completed followup: 37	N/A	Didactic lectures	10 weekly sessions, 50 minutes/ session	BMD, TCAMP	X		X

Lu and Torres-Harding 2007 [23]	U.S. Medical students	Single group before and after study	Enrolled at baseline: 469 completed followup: 469	N/A	Didactic plus experiential: Lectures; clinical case presentation; demonstrations of specific TCAM techniques; personal exposure to modalities.	Year 1 (one hour lecture), year 2 (1 hour lecture and 1.5 hours of experiential exercise), year 3 (4 hours symposium with experiential component)	Not reported		X	
Mikail et al. 2003 [24]	U.S. BMD	Single group before and after study	Enrolled at baseline: 66 completed followup: 37	N/A	Didactic; Tutorials on uses, contraindications, and drug interactions of popular herbal medicines was presented as a live session, text-only exercise, and online	Not reported	Not reported		X	

Torkelson et al. 2006 [25]	U.S. Medical students	Single group before and after study	Type: Enrolled at baseline: 26 completed followup: 24	N/A	Didactic plus experiential: Half-day TCAM site visits; small group lectures and discussion; provision of reference materials; group e-mail exchanges; and a final oral presentation and written report	15 half-day sessions	Clinicians trained in both BM and TCAM	X		

Key: BMD: biomedical doctors, TCAMP: traditional, complementary, and alternative medicine practitioners.

**Table 2:** Methodological Quality of Included Studies.

Domain	Item	Item Score	Hewson et al. 2006 [15]	Hoellein et al. 2009 [16]	Cook et al. 2007 [17]	Ashar et al. 2008 [18]	Ben-Aryee and Frenkel 2004 [19]	Ben-Aryee et al. 2006 [20]	Donald et al. 2010 [26]	Forjuoh et al. 2003 [21]	Halterman-Cox et al. 2009 [22]	Lu and Torres-Harding 2007 [23]	Mikail et al. 2003 [24]	Torkelson et al. 2006 [25]	Number of studies (%)	Mean item score (SD)	Mean domain score (SD)
Study Design																	1.79 (0.58)
	(1) Study Design:															1.79 (0.58)	
	Single group pre and post-test	1.5				x	x	x	x	x	x	x	x	x	9 (75)		
	Non randomized, 2 group	2			x										1 (8.33)		
	Randomized controlled experiment	3	x	x											2 (16.67)		

Sampling																	0.90 (0.47)
	(2) Institutions:															0.58 (0.29)	
	Single institution	0.5	x	x	x		x	x	x	x	x	x	x	x	11 (91.67)		
	Two institutions	1													0		
	More than 2 institutions	1.5				x									1 (8.33)		
	(3) Response Rate:															1.21 (0.40)	
	Response rate <50% or not reported	0.5				x						x			2 (16.67)		
	Response rate 50–74%	1							x		x		x		3 (25)		
	Response rate ≥75%	1.5	x	x	x		x	x		x				x	7 (58.33)		
Type of Data																	1.83 (1.03)
	(4) Type of Data:															1.83 (1.03)	
	Assessment by study subject	1	x				x		x	x	x	x		x	7 (58.33)		
	Objective measurement	3		x	x	x		x					x		5 (41.67)		

Validity of Evaluation Instruments' Scores																	0.36 (0.49)
	(5) Internal Structure:															0.08 (0.29)	
	Not reported	0		x	x	x	x	x	x	x	x	x	x	x	11 (91.67)		
	Reported	1	x												1 (8.33)		
	(6) Content:															1 (0)	
	Not reported	0													0		
	Reported	1	x	x	x	x	x	x	x	x	x	x	x	x	12 (100)		
	(7) Relationships to other variables:																
	Not reported	0	x	x	x	x	x	x	x	x	x	x	x	x	12 (100)	0 (0)	
	Reported	1													0		
Data Analysis																	1.38 (0.49)
	(8) Appropriateness of analysis:															1 (0)	
	Data analysis inappropriate for study design or type of data	0													0		
	Data analysis appropriate for study design and type of data	1	x	x	x	x	x	x	x	x	x	x	x	x	12 (100)		
	(9) Sophistication of analysis:															1.75 (0.45)	
	Descriptive analysis only	1					x	x						x	3 (25)		
	Beyond descriptive analysis	2	x	x	x	x			x	x	x	x	x		9 (75)		

Outcome																	1.58 (0.42)
	(10) Outcome:															1.58 (0.42)	
	Satisfaction, attitudes, perceptions, opinions, general facts	1							x	x				x	3 (25)		
	Knowledge, skills	1.5		x		x						x	x		4 (33.33)		
	Behaviors	2	x		x		x	x			x				5 (41.67)		
	Patient/health care outcome	3															

Total		18.0	13.0	13.5	13.0	12.0	9.5	11.5	9.0	9.5	10.0	9.0	11.5	8.5			10.83 (1.79)

## Appendix

### Search Strategies

Database: Ovid MEDLINE(R) 1950 to Present with Daily Update: Search Strategy:

1. exp Education, Medical, Continuing/or exp Education, Medical/or exp Education, Medical, Graduate/or exp Education, Medical, Undergraduate/(113575),
2. limit 1 to complementary medicine (3819),
3. trial.mp. Or trial.pt. or random:.mp. or randomized controlled trial.pt. or controlled clinical trial.pt. or trial.ab. or trial.mp. or trial$.mp. or groups.ab. or group$.mp. (2872432),
4. exp followup studies/or exp evaluation studies/or exp Retrospective Studies/or exp comparative studies/(2225111),
5. (compar$ or effectiv$ or multivariate or cohort or allocate$).mp. (4280712),
6. 3 or 4 or 5 (6107933),
7. 2 and 6 (1669),
8. limit 7 to (addresses or bibliography or biography or case reports or classical article or clinical conference or comment or congresses or consensus development conference or consensus development conference, nih or dictionary or directory or editorial or festschrift or guideline or historical article or in vitro or interactive tutorial or interview or introductory journal article or lectures or legal cases or legislation or letter or meta analysis or news or newspaper article or patient education handout or periodical index or portraits or practice guideline or published erratum or “review" or “scientific integrity review" or twin study or validation studies or webcasts) (212),
9. 7 not 8 (1457),
10. 10 limit 9 to humans (1267).

Database: EBM Reviews—Cochrane Central Register of Controlled Trials (4th Quarter 2010): Search Strategy:

1. ((((staff or employee* or clinician* or physician? or specialist* or practitioner*) adj (train* or course* or development or education or teach*)) or (staff or employee* or clinician* or physician? or specialist* or practitioner*)) adj (train* or course* or development or education or teach*)).mp. [mp=title, original title, abstract, mesh headings, heading words, keyword] (451),
2. limit 1 to (addresses or bibliography or biography or case report or classical article or comment or conference or congresses or consensus development conference or editorial or guideline or historical article or interview or lectures or letter or monograph or news or overall or practice guideline or “review" or “review literature" or “review of reported cases" or review, academic or review, multicase or review, tutorial or twin study) (3),
3. 1 not 2 (448).

Database: EMBASE (1980 to 2010 November 18): Search Strategy:

1. exp alternative medicine/or exp integrative medicine/or exp holistic care/or exp herb/or exp Chinese herb/or exp acupuncture/or exp chiropractic practice/or exp chiropractic/or exp manipulative medicine/or exp bodywork/or exp Chinese medicine/or exp qi gong/or exp massage/or exp ayurveda/or exp ayurveda drug/or exp traditional medicine/(100611),
2. exp EDUCATION/or exp MEDICAL EDUCATION/or exp RESIDENCY EDUCATION/or exp EDUCATION PROGRAM/or exp POSTGRADUATE EDUCATION/or exp **“**OUTCOME OF EDUCATION"/or exp CONTINUING EDUCATION/or exp CLINICAL EDUCATION/or exp VOCATIONAL EDUCATION/or exp INTERDISCIPLINARY EDUCATION/or exp CONTINUING EDUCATION PROVIDER/(738423),
3. 1 and 2 (8802),
4. Prospective study/or Intermethod comparison/or Controlled study/or (compared or compares or effectiveness or multivariate).mp. or exp followup/or Outcome research/or Treatment outcome/(5398631),
5. (cohort or compared or effectiveness or allocated or (different adj1 treatments)).tw. (2230481),
6. (cohort or groups or comparison$ or multivariate).mp. (2255439),
7. (random$ or factorial$ or placebo$ or assign$ or allocat$).mp. or Crossover Procedure.sh. or Randomized Controlled Trial.sh. or random:.tw. or clinical trial:.mp. or (random$ or placebo$).ti,ab. or controlled clinical trial$.ti,ab. (1492940),
8. Clinical trial/or randomized controlled trial/or Randomization/or Placebo/or Randomi?ed controlled trial$.tw. or Rct.tw. or Random allocation.tw. or Randomlyallocated.tw. or Allocated randomly.tw. or (allocated adj2 random).tw. or Placebo$.tw. (972747),
9. 4 or 5 or 6 or 7 or 8 (6824216),
10. 3 and 9 (2707),
11. limit 10 to human (2315),
12. limit 11 to (book or book series or conference abstract or conference paper or “conference review" or editorial or letter or note or “review" or short survey) (842),
13. 11 not 12 (1473).

Database: AMED (Allied and Complementary Medicine) (1985 to November 2010): Search Strategy:

1. Controlled study/or Randomized Controlled Trial/(1398),
2. Placebo/(509),
3. Random$.tw. (11463),
4. latin square.tw. (24),
5. crossover.tw. (525),
6. cross-over.tw. (221),
7. placebo$.tw. (2374),
8. ((doubl$ or singl$ or tripl$ or trebl$) adj5 (blind$ or mask$)).tw. (2031),
9. (comparativ$ adj5 trial$).tw. (139),
10. (clinical adj5 trial$).tw. (4227),
11. exp follow-up studies/or exp evaluation studies/or exp Retrospective Studies/or exp comparative studies/(955),
12. (compar$ or effectiv$ or multivariate or cohort or allocate$).mp. (46859),
13. exp Education medical graduate/or exp Education/or exp Education medical/or exp Curriculum/or medical education.mp. or exp Education professional/(13862),
14. 1 or 2 or 3 or 4 or 5 or 6 or 7 or 8 or 9 or 10 or 11 or 12 (54260),
15. 13 and 14 (2711),
16. limit 15 to (acupuncture or complementary medicine or “herbalism or herbal drugs" or “homeopathy or homeopathic drugs" or hypnosis or medicinal plants or traditional medicines) (63).
