# Erratum to “Complementary and Alternative Medicine Education for Medical Profession: Systematic Review”

**DOI:** 10.1155/2012/751943

**Published:** 2012-11-29

**Authors:** Nana K. Quartey, Polly H. X. Ma, Vincent C. H. Chung, Sian M. Griffiths

**Affiliations:** School of Public Health and Primary Care, Chinese University of Hong Kong, Hong Kong

In the original paper, the table describing characteristics of included studies on page 4 (Table 1) includes an error. The “Duration and frequency of intervention” of Donald et al.'s study should be “One-off intervention lasting half a day,” rather than “Not reported.”

## Figures and Tables

**Table 1:** Characteristics of included studies.

First author and publication year	Country and type of participants	Study design	Number of participants in the intervention group	Number of participants in the control group	Type of educational approach	Duration and frequency of intervention	Instructors' professional background	Outcomes reported according to Kirkpatrick level		
								2a: Attitude or perception	2b: Knowledge or skill	3: Behavioral change or intention to change
Donald et al. 2010 [26]	UK medical students	Single group before and after study	Enrolled at baseline: 40 Completed followup: 29	N/A	Experiential: personal exposure to TCAM modalities during placement	One-off intervention lasting half a day	TCAMP	X		

